# Kindness in British communities: Discursive practices of promoting kindness during the Covid pandemic

**DOI:** 10.1177/09579265221148691

**Published:** 2023-01-10

**Authors:** Jilan Wei

**Affiliations:** Changzhou Institute of Technology, China; University of Sussex, UK

**Keywords:** Community, corpus, critical discourse analysis, inclusion, kindness, pandemic, support

## Abstract

This research adopts Critical Discourse Analysis as a perspective to explore how kindness was expressed and promoted in university communities and city communities from January to March in 2020 when the Covid pandemic broke out in the UK and provide a window on British culture in which kindness was expressed and promoted through discourse. It combines a qualitative method with a corpus-based quantitative method. It is found that kindness was meant for providing support and showing compassion and inclusion to community members and that strategies in lexis, syntax and metaphor can reproduce or resist the expression and promotion of kindness in communities. During the pandemic, the intentional kindness expressed by community authorities was respect of diversity rather than inclusion of different values or ethnicity and no substantial support was provided to vulnerable members even though authorities were trying to impress the public by claiming that they were making constant efforts to support the community. Case studies revealed that we should caution against the use of passivation and the pronouns like *they*.

## Introduction

This research adopts Critical Discourse Analysis to describe how community authorities intended to express and promote kindness through discourse to the target audience such as international students (e.g. students from China) and East Asian communities at the initial stage of the Covid pandemic. It concentrates on the linguistic strategies adopted by authorities for building an inclusive and supportive community among people with diverse backgrounds and concerns in light of the dynamics of the pandemic. This work can therefore reveal how the value of kindness is deployed by community authorities when public health and safety is being challenged.

Community, traditionally, was defined based on social relations and interaction in a locality. With modernisation, urbanisation and globalisation, ‘community without propinquity’ (Webber, 1964) came into being and community is formed when individuals pursue high levels of interaction, common interests, identity and shared values (Mannarini and Fedi, 2009). It is suggested that *community* be defined along three dimensions: ecological (space, time), social structural (networks of institutions and interaction) and symbolic cultural (identity, norms and values) dimensions (Hunter, 2018).

In view of the heterogeneity of *community*, it is argued that *community* is defined not as a group of co-occurring populations, but as groups of co-occurring individuals of different populations (Looijen and Van Andel, 1999: 218). In this view, a group of individuals may be labelled as members of different communities. For example, international students may be part of the local (i.e. a city/town/village) community if they live off-campus as well as retaining their university community membership. Likewise, students from China in the UK not only belong to their host university community, but may also obtain membership in the East Asian community in that area.

The varied interpretations of *community* consistently point to the significance of cultivating sense of belonging, for which the concept of kindness is of great use. Kindness describes any voluntary act that protects or benefits others and is not driven by external rewards or punishments (Eisenberg et al., 2006) but is motivated by compassion or concern (Peterson and Seligman, 2004). Acts of kindness are ‘essentially unobligated, often emotionally complex and always deeply social’ (Anderson and Brownlie, 2019: 12).

Recent studies show that adolescents think kind acts involve 10 themes: emotional support, proactive support, social inclusion, positive sociality, complimenting, helping, expressing forgiveness, honesty, generosity, formal kindness such as fundraising and volunteering (Cotney and Banerjee, 2019); and that university students instantiate kindness by being polite, showing care and concern, being selfless, being self-aware or having a positive attitude, helping, improving other’s lives, being inclusive, nice/friendly, showing respect and following the ‘golden rule’ (Binfet et al., 2022: 448). Both groups include helping and being inclusive (social inclusion) as kind acts and believe being kind means being generous or selfless. However, it is claimed that kindness is not ‘a selfless helping’, but how help is offered, that is, ‘with gentleness, respect, amiability and concern’ (Faust, 2009: 297).

Kindness is lubricative (as a ‘social lubricant’, Binfet et al., 2022: 444) as well as reciprocal and contagious (susceptible to ‘social contagion’, Tsvetkova and Macy, 2014). The beneficiaries of kind acts are likely to give a helping hand to others (Fowler and Christakis, 2010) out of gratitude (Bartlett and DeSteno, 2006) and elevation (an emotional response to witnessing acts of virtue or moral beauty) (Algoe and Haidt, 2009: 106). In this way, kindness promotes altruism and contributes to building social connection and cultivating sense of belonging.

Kindness became a catchphrase of British community communication during the coronavirus outbreak when the public health was challenged and so many people suffered. In this research, what is focused on is the kindness expressed and described through public discourse during the pandemic.

## Background of the study

The Covid-19 pandemic has been one of the biggest challenges to human survival. China was the first country suffering greatly from the Covid-19 coronavirus in the world. In the UK, March witnessed the rapid spread of the virus, therefore the first national lockdown was put into effect on 23 March 2020 to mitigate the worsening epidemic situation. In view of the highly infectious virus, all of the students, including international students, were advised to go back home because in-person teaching was terminated and they were put at great risk of infection in their journey. What’s worse, international students from China and other residents with East Asian backgrounds who stayed in the UK were subject to racism and discrimination during the first wave of the disease. Some were assaulted and hurt physically and mentally, especially when they were wearing face masks (Lim et al., 2022: 20). Authorities in university and local communities recognised that these vulnerable members were in need of particular care and support.

## Data and methodology

During the Covid-19 crisis, through emails and statements, community authorities have disseminated great amounts of pandemic information to members in the name of kindness, with the intention of protecting the public health and safety. Based on official emails and public statements collected from four universities and three city councils in England between 23 January (first day of Wuhan lockdown) and 23 March 2020 (first day of UK lockdown), two corpora (University Corpus and City Corpus) were built (see Table 1). These emails and statements were official in that they were released from a vice-chancellor, a provost and/or a registrar or a council leader to the general public, that is, the students and staff in a university or municipal community.

**Table 1.** Corpus details.

Corpora	Words	Communities	Emails	Statements
University Corpus	63,115	UCL	0	8
		LU	5	3
		UY	4	10
		US	8	8
City Corpus	19, 557	LCC	0	4
		YCC	0	24
		BHCC	0	8

The four British universities were selected for this research because they have a high percentage of international students: University College London (UCL; 54.3%), Lancaster University (LU; 29.8%), University of York (UY; 14.1%) and University of Sussex (US; 26.7%). Moreover, UY and US are the universities with confirmed cases of Covid found in February 2020. Among the four universities, UY and LU are located in the north, while US and UCL in the south. The three city councils chosen for the research are Lancaster City Council, York City Council and Brighton and Hove City Council because they are the communities to which three of the universities belong. Camden Council, to which UCL is affiliated, is absent because no pertinent public statements or emails were available either on its website or on that of London Councils. Despite the differences in scales, members, locations and goals, the chosen communities have members from different ethnic groups, with different cultural values or beliefs. In this context it is meaningful to examine how kindness was adopted by authorities for exercising control and protecting public safety and wellbeing during this health crisis.

The perspective adopted for the research is Critical Discourse Analysis (known as CDA). According to Van Dijk (1993: 250), CDA studies ‘the relations between discourse structures and power structures more or less directly’. Between discourse and dominance, social cognition should be taken as the necessary theoretical (and empirical) ‘interface’. Ideologies are the ‘fundamental social cognitions that reflect the basic aims, interests and values of groups’ (Van Dijk, 1993: 258), ‘such as their identity, tasks, goals, norms, values, position and resources’ (Van Dijk, 1995: 18). They ‘allow members of a group to organise (admission to) their group, coordinate their social actions and goals, to protect their (privileged) resources, or, conversely, to gain access to such resources in the case of dissident or oppositional groups’ (Van Dijk, 1995: 19). In order to enact power, social actors who have privileged access to the discourse may manifest the dominance through context control as well as in some subtle and unintentional ways, such as lexical or syntactic style, rhetorical devices and local semantics. CDA therefore focuses on the discursive strategies used for legitimating control, or otherwise naturalising the social order, and especially relations of inequality (Fairclough, 1985). Situated in public health communication, CDA is of great value in showcasing how community authorities exercise control through discourse and how public discourse maintains or resists the relations of dominance and inequality.

The discourse analysis is corpus-based. With the help of Sketch Engine, corpus data is examined in terms of frequency, collocation, wordlist and concordance (including CQL, Corpus Query Language). These corpus analysis techniques are adopted for disclosing the contextual meaning of kindness and the discursive strategies for expressing or promoting kindness in university and municipal communities in times of health crisis. Furthermore, case studies are conducted with regard to wearing masks and returning home in an attempt to demonstrate what linguistic strategies better express and promote kindness within communities.

## Meaning of kindness in community

Language is viewed as an ideological phenomenon, because ideologies can be encoded and communicated through language (Volosinov, 1973). Van Dijk (2012) echoed this by asserting that ‘discourse plays a fundamental role in daily expression and reproduction of ideologies’ (p. 3). Discourse can either contribute to the ‘enactment of dominance in text and talk in specific contexts’ or have impact on ‘the minds of others’ (Van Dijk, 1993: 279). It is suggested that in doing discourse analysis, primary attention should be paid to the discursive ‘properties that express or signal the opinions, perspective, position, interests or other properties of groups’ (Van Dijk, 1995: 22), because all of these are included in ideology which any group may develop in order to cultivate ‘loyalty, cohesion, interaction and operation of its members’ (Van Dijk, 2011: 380). As part of group ideologies, kindness is explicitly or implicitly expressed in community communication. What type of kindness is stressed and pursued, and how effectively kindness is expressed and promoted can be tracked in detailed discourse analysis.

Kindness has two occurrences in University Corpus and one in Community Corpus, as shown below in Examples 1–3. Coordination is adopted for the specification and emphasis of kindness in the context. According to Lang (1984: 19), a coordinate structure is formed based on the hypothesis that conjuncts meet a set of conditions on their structural homogeneity. Conjuncts have two possible relations: compatibility (i.e. semantic non-distinctness, semantic inclusion and mutual independence) and incompatibility (contrariness and contradictoriness). Obviously, coordination used here illustrates the semantic inclusion between *kindness* and the three words *support*, *compassion* and *inclusion*.

1. Cllr Keith Aspden, Leader of City of York Council, said: It’s now five days since the Covid-19 COBRA meeting was called, and **we have been overwhelmed** by the **kindness and offers of support** from York’s residents and businesses. (City Corpus)

In Example 1, using coordination, authorities have marked the importance of support from residents and businesses as an act of kindness in community. In addition, exclusive *we* and passive voice are used to highlight authorities’ recognition of the acts of kindness and the great difference the kind support has made in community. *We* represents a group or an establishment with the speaker as a central or defining member (Wortham, 1996: 333) and it is divided into inclusive *we* and exclusive *we* (Daniel, 2005). Inclusive *we* signifies the speaker, the group or organisation he or she speaks on behalf of and the audience, while exclusive *we* doesn’t refer to the audience. The purposeful use of exclusive *we* displays their authoritative stance in recognising and advocating kindness in communities.

2. **It is** especially **importantthatwe** all show **kindness and compassion** to **our** students who are from China, who will be finding this a difficult start to the year – and **we** are particularly concerned at making sure **they** are OK. (University Corpus)

In Example 2, kindness means compassion for Chinese students in the community. The sentence pattern *it is important that. . .* allows authorities to adopt an objective perspective to make a request. What should be noted here is that compassion is different from kindness because compassion prioritises the experience of those who witness suffering whereas kindness is defined largely by the beholder or recipient rather than the giver. Moreover, compassion grows out of privilege and reaffirming hierarchies and inequalities (Spelman, 2001). Here, compassion is advocated as a particular way to show kindness, but it reveals the inequality between members and highlights the superiority of the *we*-group.

Additionally, in contrast to the inclusive *we* (*all*) and *our* which contribute to solidarity, the pronoun *they* reveals that Chinese students have been excluded from the university community. Van Dijk (1995) points out that ‘representation of ideologies are often articulated along an *us* vs *them* dimension’ (p. 22) and the use of *us* and *them* entails the positive and negative evaluations (Van Dijk, 1993: 264). Obviously, *they* is a sign of conscious or subconscious distancing, though students from China are marked as *our* group to whom great concern is expressed in this instance. Authorities othered the Chinese group of members when they were calling on members to show compassion.

3. The time has **never** been **more** important to demonstrate **our** values of **kindness and inclusion** – and to take action if **we** see anyone being treated in a harmful way. (University Corpus)

Example 3 shows that inclusion is advocated as the third specification of kindness in community. Equating kindness with inclusion, authorities intended to deliver the guidance on stopping racism and discrimination within the community. Similar to Example 2, the first-person pronouns *we* and *our* are purposefully used here. As the modifier of the noun *values*, *our*, *which* is related to inclusive *we*, that is, the audience is included, enables authorities to align members to the shared values and cultivate a sense of community. While exclusive *we* is employed to depict authorities’ agency in spotting the discriminatory behaviours, authorities’ agency hasn’t been explicitly represented in the specific response to racism and discrimination. Instead, the inanimate *time* is topicalised so as to collocate with the infinitive *to take action*. The pattern *the time has never been more important to do* . . .is used to emphasise the urgent importance of showing inclusion at that point of time. It conveys authorities’ stronger desire to advocate inclusion in community as compared to the pattern *it is important to do. . .*.

In these three examples, coordination helps define kindness as *offers of support compassion* and *inclusion* respectively. Example 1 shows the support from residents and businesses in city community has won acclaim from authorities as an act of kindness. Examples 2 and 3 indicate that compassion and inclusion have been highlighted in view of the needs of the highly vulnerable groups in communities: students from China and Asian communities.

## Expression and promotion of kindness in community

Having analysed what kindness has meant in community during the pandemic, we now concentrate on how kindness was promoted when anybody could pose risks to public safety due to the highly contagious virus and when racism and abuse took place in communities.

### Showing inclusion

To better understand kindness in community, we can pay our first attention to how community is defined. Adjectives have the inherent function of characterising a person or an object (Dixon, 1999). As attributive adjectives serve as pre-head internal dependent (i.e. part of a nominal) in the structure of the NP (Pullum and Huddleston, 2002: 528) and they can add some evaluative or descriptive information to the modified nouns, we examine the attributive adjectives of *community* to illustrate how community was described and evaluated by authorities.

**Figure 1.**
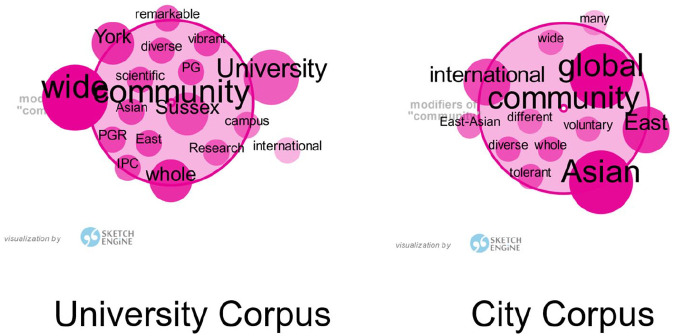
Frequencies of attributive adjectives of *community.*

As Table 2 shows, *wide*, *whole*, *South/East Asian*, *diverse* and *international* (highlighted) appear in both corpora. This suggests that in the surveyed communities diversity and solidarity are the core pursuit and South/East Asian communities have been brought in focus in community communication. In University Corpus, *wide* and *university* are used more frequently while in City Corpus *global* and *Asian* have higher frequency, as shown in Figure 1 (where the darker and larger a word is, the more frequently it appears in the corpus). This indicates both university communities and city communities paid close attention to diversity and inclusion.

4. The University is **a global** community, with one in every four people on **our** campus (both staff and students), coming from outside the UK. We value, celebrate and are proud of **our inclusive and diverse** community. (University Corpus)

**Table 2.** Attributive adjectives of *community*.

Noun	University Corpus	City Corpus
Community	***wide***, ***whole***, *York*, *university*, *remarkable*, *PG Research*, *IPC*, ***South/East Asian***, *vibrant*, *PGR*, *scientific*, ***diverse***, *Sussex*, *campus*, ***international***	*global*, ***South/East-Asian***, *Asian*, ***international***, *tolerant*, ***diverse***, ***whole***, voluntary, different, ***wide***

Concordance analysis can provide us with a wider view about what is advocated within communities. Example 4 emphasises the importance of inclusion and diversity in university communities by collocating *community* with *global*, *diverse* and *inclusive*. Additionally, the inclusive *we* and its variant *our* were intended for uniting members to show inclusion.

5. I’ve spoken to people from **Asian communities** about concerns around the origin of the virus being in China and how this could impact negatively on their community and businesses. I want to **reassure** our **international communities** that **you**’re a welcome and valued part of **our city**’s rich **fabric**. Brighton & Hove has long prided itself on being **a fair and inclusive place** to live, work and visit, for people across Britain and beyond. (City Corpus)

In Example 5, using the pronoun *you* and the verb *reassure*, the city authority was trying to show kindness directly to international communities. The impersonal reference of *you* embodies a sense of informal camaraderie and a sense of universality (Kitagawa and Lehrer, 1990: 742). What is worth noting here is the adoption of the metaphor in which *city* is likened to *fabric* to stress the importance of diversity in the community. In order to promote kindness within the city, the authority mentioned the concerns expressed to Asian communities about the negative impact of the virus on them. By contrast, inclusion was shown to international communities which may include but is not limited to Asian communities. This subtle shift is likely to help authorities articulate their vague stance in supporting Asian communities and avoid any challenge from local communities who may have bias towards Asian communities.

Although authorities claimed that communities were inclusive and diverse, racism, abuse and discrimination did occur during the pandemic and it had severe social impacts. At the initial stage of the pandemic, in the UK, ‘maskaphobia’ (dubbed by *The Guardian*) prevailed and wearing a surgical mask in public, especially if you looked East Asian, could even invite racist attacks (Weale, 2020).

6. We have been saddened by reports of racism from a minority of the public. **This type of behaviour** has no place in York or anywhere else. As a city of sanctuary, we respect and welcome cultures and communities from across the world. (City Corpus)

When racism and discrimination took place in British communities, authorities displayed unfavourable attitudes in different ways. As illustrated in Examples 6–8, city authorities have shown their different stances. In Example 6, authorities expressed their sadness about reports of racism and announced the ban for racism in the community. Nonetheless, a passive structure is used for emotional expression and an active voice is adopted for announcing the ban. Although passivation implicates authorities’ negative attitudes towards racism, the ban with *this type of behaviour* as the topicalised subject doesn’t show any specific actions would be taken to stop racism. Instead, what has been advocated here is just formalistic respect of cultural diversity.

7. The Vice-Chancellor of Lancaster University, Professor Steve Bradley, said: ‘I have made it clear to **our** students that if **they** are subject to any form of racism or discrimination, **they** must report it immediately **and we** will take action. It’s very important at this difficult time that **they** feel **they** have **our** support and understanding’. (City Corpus)

In Example 7, authorities are trying to encourage students to report racism and discrimination by quoting a professor. Coordination used in this instance implies that no action would be taken if students who were subject to any form of racism or discrimination didn’t report. Moreover, the frequent use of *we*, our, *they* clearly shows the distinction between in-groups and out-groups in the community.

8. Councillor Dr Erica Lewis, leader of Lancaster City Council, said: ‘Our district is stronger for being a diverse and tolerant community. I am deeply concerned to hear that **any residents** has been subject to racist abuse, and condemn this behaviour. At a time when people are worried about friends and family, it is important that we show our support for **those members of our community**, and our concern for **everyone** infected and affected, particularly communities in China and South East Asia where the greatest impacts are being felt’. (City Corpus)

In Example 8, authorities don’t use the pronoun *they* or any of its variants to refer to the people affected by racism. Conversely, *any residents*, *those members of our community* and *everyone* are adopted to include the vulnerable group in the community. In this way, support and inclusion are well expressed and advocated.

9. London is a diverse city which is home to a vibrant community from around the world. But like any major city, deplorable incidents like this can occur. During the current outbreak of coronavirus, we are very proud of the way in which our community has responded calmly and is supporting each other. We want to make clear that **abuse, racism and hate speech** have no place at UCL. (University Corpus)

Examples 9–11 can depict how university authorities positioned themselves in dealing with racist abuse and hate speech during the pandemic. Similar to Example 6, Example 9 topicalises *abuse*, *racism* and *hate speech* to inform the members of a relevant ban. What’s interesting here is authorities’ explanation for the happening of the saddening incidents: London is a diverse community where the incidents are bound to happen. This naturalises the occurrence of racism, abuse and hate speech in London though it claims the rejection of the deplorable incidents at UCL.

10. There have been some cases in the news of people experiencing bullying and abuse related to coronavirus. We would like to remind everyone that UCL and Students’ Union UCL **do not tolerate** bullying or abuse of any kind, including racial discrimination and hate speech. (University Corpus)

11. Unfortunately, there have been some shocking media reports about abuse and racism at other universities linked to the current coronavirus situation. This behaviour **will not be tolerated** at Lancaster University. We **encourage** anyone experiencing or witnessing abuse or inappropriate behaviour to report it to the University. (University Corpus)

Contrary to Example 9, Example 10 adopts an active voice to show an institutional stance in dealing with bullying or abuse: *not tolerate*. Example 11 demonstrates university authorities’ unfavourable attitudes towards virus-related racial discrimination. On the one hand, they displayed their intolerance of abuse and racism implicitly through passivation and topicalisation (*This behaviour will not be tolerated*). On the other hand, they actively encouraged reporting.

The examples from the two types of communities consistently show that authorities suppressed their agency in tackling virus-related abuse and discrimination and they encouraged or requested members to report for getting support. In community communication, what we can see is the promotion of support, diversity and inclusion, but we can’t see description of any specific action to prevent the happening of racism and discrimination. In addition, when delivering regulations, university and city authorities were aware of their domination in the public communication and they favoured the patterns such as *it is important that. . .* and *we want/would like to remind/make clear that. . .*. Further examination tells us these two patterns have different semantic prosodies. Subordination in the pattern *It is important that. . .*carries positive prosody and expresses the shared values in communities, while what is subordinated to *we would like to remind/make clear that. . .*connotates negative profiles and conveys disagreement. Despite the different semantic prosodies, the two types of sentence patterns were intended for promoting kindness, respect and inclusion within communities, whereby authoritative and dominant position got guaranteed and maintained.

### Offering support

As a word, *support* has two parts of speech. As a verb, in University Corpus it occurs 106 times (frequency per million words: 1500.63), while in City Corpus it has 61 occurrences (frequency per million words: 2751.84). As a noun, it has 161 instances (frequency per million words: 2279.26) in University Corpus and 55 instances (frequency per million words: 2481.17) in City Corpus. In University Corpus it is used more frequently as a noun, while in City Corpus it has higher frequency as a verb. Whether it is a noun or a verb, *support* has higher relative frequency in City Corpus than in University Corpus. However, in both corpora it ranks 22nd or higher (its rankings are highlighted in bold) in the word frequency list as each part of speech (see Table 3).

**Table 3.** Use of *support* in the corpora.

Parts of speech	Ranking	Top list
Verb	**Top 17** in University Corpus	*be*, *have*, *do*, *please*, *take*, *follow*, *need*, *work*, *continue*, *return*, *contact*, *provide*, *advise*, *include*, *make*, *use*, ***support***
	**Top 7** in City Corpus	*be*, *have*, *work*, *include*, *do*, *continue*, ***support***
Noun	**Top 13** in University Corpus	*student*, *advice*, *coronavirus*, *university*, *staff*, *UCL*, *health*, *information*, *travel*, *home*, *UK*, *Covid-19*, ***support***
	**Top 22** in City Corpus	*health*, *city*, *advice*, *council*, *people*, *public*, *York*, *coronavirus*, *information*, *England*, *case*, *service*, *government*, *Covid-19*, *business*, *child*, *NHS*, *school*, *contact*, *community*, *home*, ***support***

In order to figure out how community authorities offered their support and help to members, we can take a close look at the use of modal verbs, as they carry important information about the sender’s attitude to the message and other interpersonal meanings (McCarthy, 1991).

12. We will let you know more details **as soon as we can**, but please note that this may take several weeks. (University Corpus)

13. We are grateful for all the support that so many people at Sussex have offered to our students and staff from China. Please know that the University is here to help and support you **however we can**. (University Corpus)

14. It is abundantly clear that we are living in unprecedented times and dealing with exceptional circumstances. As a city, we must find our resolve and do **everything we can** to support those who need our help the most. (City Corpus)

15. Council staff have been adjusting and re-focusing their efforts to support our communities over the coming months, particularly to ensure our frontline services can continue to operate to keep the city moving. I would like to thank the fantastic response of our staff during this crisis, and we will continue to do **what we can** to support residents, businesses and communities across the city. (City corpus)

*Can* is a modal verb employed to express ability, possibility and permission. It is used for making offers and requests. Collocating with the pronoun *we*, it reflects that authorities were able or likely to support and serve the members during the health crisis. Such expressions as *everything we can*, *however we can* and *all we can* are constantly used to demonstrate authorities’ ability and resolve to support and protect community members. In Example 12, *we can* appears in an adverbial clause and displays authorities’ active role in information service. The second conjunct in the example further reveals that *several weeks* was marked ‘soon’ and authorities were actually not so capable of providing more information. Example 13 also depicts the use of *can* in an adverbial for demonstrating authorities’ sincerity and determination involved in helping and supporting members. On the other hand, *can* which appears in *everything we can* and *what we can* may contribute to constructing authorities’ active image in community support, illustrated in Examples 14 and 15.

*We can* has 64 and 21 cases in University Corpus and City Corpus respectively. As Table 4 shows, in total, the subordinated *we can* accounts for 36.0% and 42.9% respectively in the corpora. This means that *can* was favoured by authorities for expression of attitudes rather than description of actions in response to the pandemic.

16. Please refer to this page for accurate and updated advice about the coronavirus and UCL’s response. **We will** update this page regularly with more information as it becomes available. (City Corpus)

**Table 4.** Use of *we can* in subordination.

Patterns	University Corpus		City Corpus	
	Frequency	Percentage (%)	Frequency	Percentage (%)
as. . .as *we can*	6	9.4	1	4.8
everything/all *we can*	11	17.2	5	23.8
however *we can*	3	4.7		
what *we can*	3	4.7	3	14.3
**Total**	23	**36.0**	9	**42.9**

*Will* enables authorities to demonstrate their determination and capability in responding to the health crisis. It can point to either volition or future prediction, or both. Normally it is used with a human subject and it has volitional element though it can also be a future marker (Aijmer, 1985). *Will* mainly collocates with the animate subjects *we* and *you*. In Example 16, *we will* signals a volitional action in the future. Just because of its vagueness, *will* becomes preferable to other modal verbs. It has 744 occurrences in University Corpus and 118 in City Corpus, collocating with *we* 148 and 33 times respectively. It is followed by *be* and *continue* more frequently in both corpora.

Next, we turn to the application of tense or aspect to showing support. Semantically, aspect involves the boundedness of events (Radden and Dirven, 2007). Progressive aspect marks an event which is conceptualised as unbounded from ‘inside’ the situation where it is occurring. In addition, progressive aspect contributes to expressing ‘greater immediacy’ and implicating the potential continuation of a situation unless impeded (Radden and Dirven, 2007: 178). In contrast, perfect aspect is used for an event which is bounded from the ‘outside’. Present perfect aspect means that an event has an endpoint which is the conceptualiser’s *now*. Different from present perfect aspect, present perfect progressive aspect highlights the continuous or interrupted duration of a situation and it has inferential (describing ‘the inferred state following an anterior unbounded event’) and continuative (stressing ‘the durational phase of a situation’) functions (Radden and Dirven, 2007: 217).

17. **We will be providing** a remote and reduced operating model for accessing services and facilities on campus. (University Corpus)

18. **We are working** closely with partners about how to best support those at risk at food poverty and who claim free school meals, and further guidance will be issued. (City Corpus)

19. Since the outbreak of the Coronavirus **we have been making** every effort to safeguard our staff and students around the world. (University Corpus)

In Example 17, a declarative sentence adopts the future progressive tense and helps university authorities to display their agency in responding to the pandemic in the future. In Example 18, a declarative sentence in the present progressive tense describes the event where city authorities make continuous efforts in providing support for members. Example 19 uses the present perfect progressive tense to show the durational phase of authorities’ efforts in protecting the community.

No matter in what aspects, progressive tense effectively reflects authorities’ ongoing attempts to support and serve members when public health is under threat. And frequency counts show that *we are doing* (“we” “be” [tag= “V.*”]) enjoys the most popularity among the three patterns, illustrated in Table 5. This suggests that authorities were trying to impress the public as an organisation that was making constant efforts to support members and protect the public health and safety.

**Table 5.** Frequencies of three patterns with *we.*

Patterns	University Corpus	City Corpus
“we” “be” [tag= “V.*”]	80	41
“we” “will” “be” [tag= “V.*”]	14	6
“we” “have” “be” [tag= “V.*”]	14	13

## Two case studies on kindness expression

Having explored how kindness is expressed and promoted through discourse in community, we need to check if there is any discursive evidence for expression of and deviation from kindness with respect to specific themes.

### Wearing masks

With regard to wearing masks in the pandemic, opinions and practices may vary from member to member in community. In public statements *they* is constantly used as the synonym of out-groups and negative attitudes are attached to it. In Example 20, face-mask group has been marked as out-group by means of the pronoun *their*. The initiative was intended to advocate respect and solidarity within community, but it negated the protective practice which is conventional and acceptable in Asian communities by using the evidential structure *there is little evidence that. . .*. Consequently, the supportive and kind proposal fails to meet the psychological needs of face-mask group, let alone cultivating their sense of community.

20. Should **I** wear a protective face mask?

Advice from the NHS and Public Health England states that following hygiene precautions such as thoroughly washing hands with soap and water, covering your mouth and nose with a tissue if you cough or sneeze, and keeping surfaces clean, are the best ways to avoid catching or spreading the virus. **There is little evidence that** masks are effective in preventing the spread of the virus.

However, we know that **some people** may choose to wear face masks to protect themselves and others from possible infection. **It is importantthat** we recognise **their** decision and we encourage our community to be as supportive of each other as possible. (University Corpus)

In addition, Example 20 exploits the sentence pattern *it is important that. . .* for delivering the authoritative or persuasive message in community. This pattern, together with its variant *it is important to do. . .*, is often adopted for formal suggestion or persuasion because it enables authorities to distance themselves from the current situation so as to make a suggestion or guidance as objectively as possible.

21. Should **I** wear a mask?

**Some of our community** choose to wear face masks for cultural, social and personal reasons. The wearing of a face mask is not necessarily a sign that **the wearer** is ill with a cold, flu or any other virus. Public Health England do not stress the need to wear a face mask but instead issue this advice to prevent sharing germs. As such, the University will not be asking anyone to wear masks or supplying them, but please remember that **some people** choose to. (University Corpus)

Conversely, Example 21 can be taken as a better choice for authorities to resolve the dispute of wearing masks in communities. By using the phrase *some of our community*, authorities have skilfully included face-mask group in community and, to some degree, successfully nurtured their sense of community. Then adopting the nominalised subject (*the wearing of a face mask*) and the neutral signifier (*the wearer*), authorities were trying to eliminate misunderstanding about mask-wearing in community. By quoting Public Health England for the use of face masks, authorities eventually displayed their mediating stance: mask wearing is not an obligation but a personal choice and it should be respected.

What is worth mentioning here is that when Covid-19 situation became serious in the UK, more communities started to use *I* instead of *you* to refer to the audience (mainly students and staff) so as to give the contextually dependent guidance directly and effectively. As can be seen in Examples 20 and 21, the pronoun *I* signifies a perspective change in providing specific guidance and moral support. It subtly converts a formal dialogue to an intimate soliloquy so as to facilitate the information delivery and promote kindness expression.

### Returning home

Regarding public guidance or advice, authorities tended to adopt passive voice to indicate its formality and objectivity, as shown in Example 22.

22. I want to go home because of the coronavirus outbreak. What is UCL’s policy on this?

On 17 March, UCL announced that **most university buildings will be closed** by Friday 20 March and **students are strongly advised** to return home wherever possible. (University Corpus)

However, passivation implies little attention is paid to the emotional needs or mental health of members at this unprecedented time. The advice on returning home cannot gloss over the unequal power relations between university authorities and students. It also effectively suppresses the agentic role of authorities in urging students to leave the campus. In this way, no one should take on the responsibility of publicising the guidance.

23. **We strongly recommend** that it is in **your** interest to make **your** travel plans as soon as practically possible if **you** do wish to return home. (University Corpus)

In public discourse, personal pronoun *you* contributes to defining the target audience and constructing a dialogic communication model. As shown in Example 23, using the pronouns *you* and its variant *your* to refer to students, authorities stated straightforwardly their advice about returning home. Kindness and concern were intended to express by means of the sentence pattern *it is in your interest to do. . .*. Nevertheless, activation increases the level of imposition and deviates a little from authorities’ kind intention. It seems to be incompatible with the British culture which highlights personal autonomy and negative politeness. However, it is likely to demonstrate an affective stance and render the persuasion emotional and appealing.

## Discussion and conclusion

Kindness is not only a personal virtue, but also a social lubricant and a catalyst for altruism. By expressing and promoting kindness through discourse, community authorities intended to cultivate members’ sense of community and maintain their dominant position. This research explores how kindness was expressed verbally to the vulnerable group of people in community, that is, students from China and East Asian communities, during the health crisis.

Detailed discourse analysis has uncovered what kindness was meant and promoted by authorities in British university communities and city communities during the Covid pandemic. It is found that kindness was instantiated by providing support to community members and showing compassion and inclusion to members with different cultural backgrounds. This justifies young people’s interpretations of kind acts as helping and inclusion (Binfet et al., 2022; Cotney and Banerjee, 2019).

Kindness, especially adolescent kindness, falls into three categories: random or reactionary kindness, intentional kindness and quiet kindness (Binfet and Enns, 2018). Evidently, what community authorities expressed and promoted was intentional kindness, which involved ‘planning, gathering resources, identifying recipients, scheduling, and execution’ (Binfet and Enns, 2018: 35). The intentional kindness was oriented towards Chinese students or East Asian communities and aimed at exercising control on community members and maintaining the public safety at this particular time.

Carefully choosing adjective modifiers of *community* as well as the pronouns such as inclusive *we* and impersonal *you*, authorities strived to emphasise diversity and inclusion and unite members to show kindness and respect diversity. Comparing city to fabric, authorities intended to picture metaphorically a diverse and inclusive community and advocate community diversity and inclusion.

However, we have to differentiate inclusion from diversity. Diversity and inclusion are often loosely conflated, but diversity does not generate inclusion automatically (Johnson, 2011; Sherbin and Rashid, 2017). As the functional partner to diversity, inclusion requires the shift from performative diversity (highlighting quotas and statistics as a measure of diversity) to cognitive diversity (emphasising creating space to diverse opinions and perspectives) (Brix et al., 2022: 267). During the pandemic, mask wearing has become a touchstone of community inclusion. In using the pronoun *they* and its variants *their* and *them*, authorities consciously or accidentally excluded some groups (e.g. students from China or other Asian countries) from a community (e.g. university community) and negate the values or beliefs (e.g. wearing masks is protective) of some members so much so that kindness (i.e. respect and inclusion) couldn’t be expressed or promoted effectively within communities. When discrimination and racism did take place, community authorities, in most times, suppressed their agency to protect the vulnerable groups. They adopted passivation and topicalisation to subtly distance themselves from virus-related racial discrimination and take an objective stance in dealing with the unkind and inappropriate behaviours so as to mediate the complex relationships among members in a safe position. Overall, their authoritative stance in dealing with racism and discrimination was featured by reminders or warnings other than action plans. It is true that this may be interpreted as a respectful speech act which corresponds to the no-imposition British culture, but this fails to satisfy the emotional and safety needs of the target audience. In addition, in the metaphor of fabric, what was highlighted was diversity rather than inclusion because what authorities welcomed was not Asian communities, but international communities.

Kindness can also be seen in providing support. Using declarative sentences in progressive tense with different aspects, authorities underlined their continuous efforts in supporting members. Additionally, modal verbs such as *can* and *will* enable authorities to demonstrate their active role in providing support and expressing kindness. The constant use of *we can* in subordination (e.g. *everything we can*, *as soon as we can*) is conducive to displaying authorities’ volition and sincerity involved in giving support to people. However, it cannot show what specific or substantial actions authorities were able or likely to take to help the members. More evidence of attitudinal display other than action-oriented narrative can be found in the subordinated request, advice or warning in such sentence patterns as *it is important that. . ., it is important to do. . ., we would like to remind that. . .* and *we want to make clear that. . .*. The verbal kindness were intended to give support to the vulnerable group in the community. However, this British style of persuading values kindness and respect to every member, not just the vulnerable groups of people in urgent need of help and support. Therefore, it may be taken as a kind of lip service and impressed as ritualised kindness.

Just as what Jones (2021) has said, discourse analysis can ‘provide us with frameworks to notice when and how meaning is created, and sometimes to productively intervene’ (Jones, 2021: 3). Based on the two case studies, we can figure out how kindness is likely to be better expressed in discursive practice. It is suggested that we caution against the use of the third-person plural pronoun *they* and the passive structure in expressing and advocating kindness so as to well protect members’ safety and well-being during the pandemic.

This research is a tentative attempt to explore the expression and promotion of kindness within British university communities and city communities at the beginning of the pandemic from the perspective of CDA. Kindness expression and promotion has been contextualised and targeted towards some groups of members in the community communication during the Covid pandemic. This research has examined a subset of the linguistic ways to convey or advocate kindness. The next step could be to survey the attitudes of the target audience to the official emails and public statements and examine the effectiveness of kind expression in community in terms of the beholders and recipients.

## References

[bibr1-09579265221148691] Aijmer K (1985) The semantic development of *will*. In: Fisiak J (ed.) Historical Semantics-Historical-Word-Formation. Amsterdam, Netherlands: Mouton, pp.11–21.

[bibr2-09579265221148691] Algoe SB Haidt J (2009) Witnessing excellence in action: The ‘other-praising’ emotions of elevation, gratitude, and admiration. The Journal of Positive Psychology 4(2): 105–127. 19495425 10.1080/17439760802650519 PMC2689844

[bibr3-09579265221148691] Anderson S Brownlie J (2019) Public Policy and the Infrastructure of Kindness in Scotland. Dunfermline: Carnegie UK Trust.

[bibr4-09579265221148691] Bartlett MY DeSteno D (2006) Gratitude: Helping when it really costs you. Psychological Science 17(4): 319–325. 16623689 10.1111/j.1467-9280.2006.01705.x

[bibr5-09579265221148691] Binfet JT Enns C (2018) Quiet kindness in school: Socially and emotionally sophisticated kindness flying beneath the radar of parents and educators. Journal of Childhood Studies 42(2): 31–45.

[bibr6-09579265221148691] Binfet JT Willis-Stewart S Lauze A , et al. (2022) Understanding university students’ conceptualizations and perceptions of kindness: A mixed methods study. Journal of Further and Higher Education 46(4): 441–460.

[bibr7-09579265221148691] Brix KA Lee OA Stalla ASG (2022) Understanding inclusion. BioScience 72(3): 267–275.

[bibr8-09579265221148691] Cotney JL Banerjee R (2019) Adolescents’ conceptualisations of kindness and its links with well-being: A focus group study. Journal of Social and Personal Relationships 36(2): 599–617.

[bibr9-09579265221148691] Daniel M (2005) Understanding inclusives. In: Filimonova E (ed.) Clusivity: Typology and Case Studies of Inclusive-Exclusive Distinction. Amsterdam, Netherlands; Philadelphia, PA: John Benjamins Publishing Company, pp.3–48.

[bibr10-09579265221148691] Dixon RMW (1999) Adjectives. In: Brown K Miller T (eds) Concise Encyclopedia of Grammatical Categories. Amsterdam, Netherlands: Elsevier, pp.1–8.

[bibr11-09579265221148691] Eisenberg N Fabes R Spinrad T (2006) Prosocial development. In: Damon W Lerner RM (eds) Handbook of Child Psychology Volume 3: Social, Emotional and Personality Development, 6th edn. Hoboken, NJ: John Wiley and Sons, Inc, pp.646–718.

[bibr12-09579265221148691] Fairclough NL (1985) Critical and descriptive goals in discourse analysis. Journal of Pragmatics 9(6): 739–763.

[bibr13-09579265221148691] Faust HS (2009) Kindness, not compassion, in healthcare. Cambridge Quarterly of Healthcare Ethics 18(3): 287–299. 19460231 10.1017/S0963180109090458

[bibr14-09579265221148691] Fowler JH Christakis NA (2010) Cooperative behavior cascades in human social networks. Proceedings of the National Academy of Sciences 107(2): 5334–5338. 10.1073/pnas.0913149107 PMC2851803 20212120

[bibr15-09579265221148691] Hunter A (2018) Conceptualising community. In: Cnaan RA Milofsky C (eds) Handbook of Community Movements and Local Organizations in the 21st Century. Cham, Switzerland: Springer International, pp.3–23.

[bibr16-09579265221148691] Johnson DR (2011) Women of color in science, technology, engineering, and mathematics (STEM). New Directions for Institutional Research 2011(152): 75–85.

[bibr17-09579265221148691] Jones RH (ed.) (2021) Viral Discourse. Elements in Applied Linguistics. Cambridge: Cambridge University Press.

[bibr18-09579265221148691] Kitagawa C Lehrer A (1990) Impersonal use of personal pronouns. Journal of Pragmatics 14(5): 739–759.

[bibr19-09579265221148691] Lang E (1984) The Semantics of Coordination. Amsterdam, Netherlands: John Benjamins.

[bibr20-09579265221148691] Lim MA Li H Yu J , et al. (2022) COVID 19 incidents against Chinese and East Asian students in the UK: Safety security and communication. HERE@Manchester.

[bibr21-09579265221148691] Looijen RC Van Andel J (1999) Ecological communities: Conceptual problems and definitions. Perspectives in Plant Ecology, Evolution and Systematics 2(2): 210–222.

[bibr22-09579265221148691] McCarthy M (1991) Discourse Analysis for Language Teachers. Cambridge: Cambridge University Press.

[bibr23-09579265221148691] Mannarini T Fedi A (2009) Multiple senses of community: The experience and meaning of community. Journal of Community Psychology 37(2): 211–227.

[bibr24-09579265221148691] Peterson C Seligman MEP (2004) Character Strengths and Virtues: A Handbook and Classification. Oxford: Oxford University Press.

[bibr25-09579265221148691] Pullum GK Huddleston R (2002) The Cambridge Grammar of the English Language. Cambridge: Cambridge University Press.

[bibr26-09579265221148691] Radden G Dirven R (2007) Cognitive English Grammar. Amsterdam, Netherlands: John Benjamins.

[bibr27-09579265221148691] Sherbin L Rashid R (2017) Diversity doesn’t stick without inclusion. Harvard Business Review, 1 February 2017. Available at: https://hbr.org/2017/02/diversity-doesnt-stick-without-inclusion (accessed 10 October 2022).

[bibr28-09579265221148691] Spelman E (2001) Fruits of Sorrow: Framing our Attention to Suffering. Boston, MA: Beacon Press.

[bibr29-09579265221148691] Tsvetkova M Macy MW (2014) The social contagion of generosity. PLoS One 9(2): e87275. 10.1371/journal.pone.0087275 PMC3923723 24551053

[bibr30-09579265221148691] Van Dijk TA (1993) Principles of critical discourse analysis. Discourse and Society 4(2): 249–283.

[bibr31-09579265221148691] Van Dijk TA (1995) Discourse analysis as ideology analysis. In: Schaffne C Wenden AL (eds) Language and Pace. London: Routledge, pp. 17–33.

[bibr32-09579265221148691] Van Dijk TA (2011) Discourse and ideology. In: Van Dijk TA (ed.) Discourse Studies: A Multidisciplinary Introduction. London: SAGE, pp. 379–408.

[bibr33-09579265221148691] Van Dijk TA (2012) Ideology and Discourse: A Multidisciplinary Introduction. Available at: https://discourses.org/wp-content/uploads/2022/06/Teun-A.-van-Dijk-2012-Ideology-And-Discourse.pdf (accessed 1 July 2022).

[bibr34-09579265221148691] Volosinov VN (1973) Marxism and the Philosophy of Language: Studies in Language (trans. Matejka L Titunik IR ). London: Seminar Press Limited.

[bibr35-09579265221148691] Weale S (2020) Chinese students flee UK after ‘maskaphobia’ triggered racist attacks. The Guardian. Available at: https://www.theguardian.com/education/2020/mar/17/chinese-students-flee-uk-after-maskaphobia-triggered-racist-attacks (accessed 25 April 2020).

[bibr36-09579265221148691] Webber M (1964) Urban place and the nonplace realm. In: Webber MM Dyckman JW Fole y DL , et al. (eds) Explorations Into Urban Structure. Philadelphia, PA: University of Pennsylvania Press, pp.79–153.

[bibr37-09579265221148691] Wortham SEF (1996) Mapping participant deictics: A technique for discovering speakers’ footing. Journal of Pragmatics 25(3): 331–348.

